# Aβ1-42 Accumulation Accompanies Changed Expression of Ly6/uPAR Proteins, Dysregulation of the Cholinergic System, and Degeneration of Astrocytes in the Cerebellum of Mouse Model of Early Alzheimer Disease

**DOI:** 10.3390/ijms241914852

**Published:** 2023-10-03

**Authors:** Maxim L. Bychkov, Aizek B. Isaev, Alexander A. Andreev-Andrievskiy, Konstantin Petrov, Alexander S. Paramonov, Mikhail P. Kirpichnikov, Ekaterina N. Lyukmanova

**Affiliations:** 1 Shemyakin-Ovchinnikov Institute of Bioorganic Chemistry, Russian Academy of Sciences, 119997 Moscow, Russia; maksim.bychkov@gmail.com (M.L.B.); isaev.aizek@gmail.com (A.B.I.); a.s.paramonov@gmail.com (A.S.P.); kirpichnikov@inbox.ru (M.P.K.); 2 Moscow Institute of Physics and Technology, State University, 141701 Dolgoprudny, Russia; 3 Interdisciplinary Scientific and Educational School of Moscow University «Molecular Technologies of the Living Systems and Synthetic Biology», Faculty of Biology, Lomonosov Moscow State University, 119234 Moscow, Russia; aandrievsky@gmail.com; 4 Institute for Biomedical Problems of Russian Academy of Sciences, 123007 Moscow, Russia; 5 Arbuzov Institute of Organic and Physical Chemistry, Federal Research Center “Kazan Scientific Center of the Russian Academy of Sciences”, Arbuzov Str., 8, 420088 Kazan, Russia; kpetrov2005@mail.ru; 6 Biological Department, Shenzhen MSU-BIT University, Shenzhen 518172, China

**Keywords:** Alzheimer disease, Ly6/uPAR, Lynx1, Lypd6, SLURP-1, PSCA, nicotinic acetylcholine receptor, astrocytes, motor memory

## Abstract

Alzheimer disease (AD) is a widespread neurodegenerative disease characterized by the accumulation of oligomeric toxic forms of β-amyloid (Aβ1-42) and dysfunction of the cholinergic system in the different brain regions. However, the exact mechanisms of AD pathogenesis and the role of the nicotinic acetylcholine receptors (nAChRs) in the disease progression remain unclear. Here, we revealed a decreased expression of a number of the Ly6/uPAR proteins targeting nAChRs in the cerebellum of 2xTg-AD mice (model of early AD) in comparison with non-transgenic mice both at mRNA and protein levels. We showed that co-localization of one of them, – neuromodulator Lynx1, with α7-nAChR was diminished in the vicinity of cerebellar astrocytes of 2xTg-AD mice, while Aβ1-42 co-localization with this receptor present was increased. Moreover, the expression of anti-inflammatory transcription factor KLF4 regulating transcription of the *Ly6/uPAR* genes was decreased in the cerebellum of 2xTg-AD mice, while expression of inflammatory cytokine TNF-α was increased. Based on these data together with observed astrocyte degeneration in the cerebellum of 2xTg-AD mice, we suggest the mechanism by which expression of the Ly6/uPAR proteins upon Aβ pathology results in dysregulation of the cholinergic system and particularly of α7-nAChR function in the cerebellum. This leads to enhanced neuroinflammation and cerebellar astrocyte degeneration.

## 1. Introduction

Alzheimer disease (AD) is a neurodegenerative disease, which accounts for ~60–70% of the total cases of dementia [1]. It usually starts slowly but progressively worsens leading to impairment of cognitive function, behavioral abnormalities, and loss of indispensable body functions. The survival time from the AD diagnosis ranges from ~3 to ~7 years [2], during most of which the patient life quality is extremely hampered, so development of new therapeutic strategies is very important. However, the reasons for AD onset as well as the molecular mechanisms of the disease are still poorly studied. The most known pathophysiological features of AD are the accumulation of parenchymal amyloid beta (Aβ) plaques and the formation of intracellular neurofibrillary tangles from the tau-protein in the brain, resulting in synaptic damage and the progressive loss of cognitive function [3,4]. Aβ plaques are aggregates formed from homologous peptides which are processed from larger amyloid precursor protein (APP). APP is a transmembrane protein, which regulates synaptic transmission after cleavage of its extracellular part by α-secretase, while Aβ fibrils processed by β- and γ-secretases aggregate into insoluble complexes in the brain parenchyma. Early onset of AD is linked with mutations in three genes—the *APP* gene and the genes coding γ-secretase subunits presenilin 1 and 2 (*PSEN1* and *PSEN2*), while late AD onset is considered a multifactorial disease with a complex background [3].

The most toxic forms of Aβ are Aβ1-40 and Aβ1-42 [3]. These peptides bind to the nicotinic receptor type α7 (α7-nAChR), which is important for synaptic transmission, learning, and memory [5,6,7]. After binding to α7-nAChR, Aβ1-40 and Aβ1-42 may cause receptor endocytosis and death of the neurons [8,9]. Potentiation of α7-nAChR leads to neuroprotection; moreover, agonists of α7-nAChR can activate survival pathways in neurons, protecting cells from toxicity mediated by the Aβ1-42/α7-nAChR interaction [10,11]. Recently, it was shown that motor impairment and attention disorders in AD pathogenesis are accompanied by a reduction in the volume of the cerebellar cortex and gray matter in the anterior lobe of the cerebellum [12,13,14]. However, the role of α7-nAChR in regulation of early onset of AD in the cerebellum presently remains unclear.

The endogenous proteins from the Ly6/uPAR family (Lynx1, Lynx2, Lypd6, Lypd6b, Ly6e, Ly6h, Ly6g6e, PSCA, SLURP-1, SLURP-2) modulate nAChR’s function [15,16,17,18,19,20,21,22,23,24,25]. Expression of Lynx1, an α7-nAChR positive modulator in the brain [17,26], is down-regulated in the frontal cortex of 3xTg-AD mice with β-amyloid and tau pathology [27]. Diminished expression of Lynx1 and Lypd6b [28] was recently reported in the hippocampal neurons of 2xTg-AD mice (a model of the early stage of AD with hyperexpression of mutant presenilin-1 (PS1-dE9) and chimeric APP with Swedish mutation K595N/M596L [29]). In contrast, expression of PSCA is increased in the cortex of patients with AD [30]. Thus, dysregulation of expression of endogenous nAChR modulators from the Ly6/uPAR family in the brain could be associated with AD onset. However, the influence of the β-amyloid pathology on the expression of the Ly6/uPAR proteins in the cerebellum and how this can affect the cholinergic system and other processes in the AD brain has not been studied yet.

Here, we revealed the changes in expression of a number of the Ly6/uPAR proteins targeting nAChRs in the cerebellum of 2xTg-AD mice both at mRNA and protein levels. We showed that co-localization of Lynx1 with α7-nAChR was diminished in the vicinity of the cerebellar astrocytes of 2xTg-AD mice, while co-localization of Aβ1-42 with this receptor was increased. Moreover, the expression of anti-inflammatory transcription factor KLF4 was decreased, while expression of an inflammatory cytokine TNF-α was increased. Based on the obtained results we proposed the mechanism by which the changed expression of the Ly6/uPAR proteins results in dysregulation of the cholinergic system, and particularly of α7-nAChR function in the brain. This, in turn, results in activation of neuroinflammation and astrocyte degeneration in the cerebellum of 2xTg-AD mice.

## 2. Results

### 2.1. Expression of the Ly6/uPAR Proteins Is Changed in the Cerebellum of 2xTg-AD Mice

**Figure 1:**
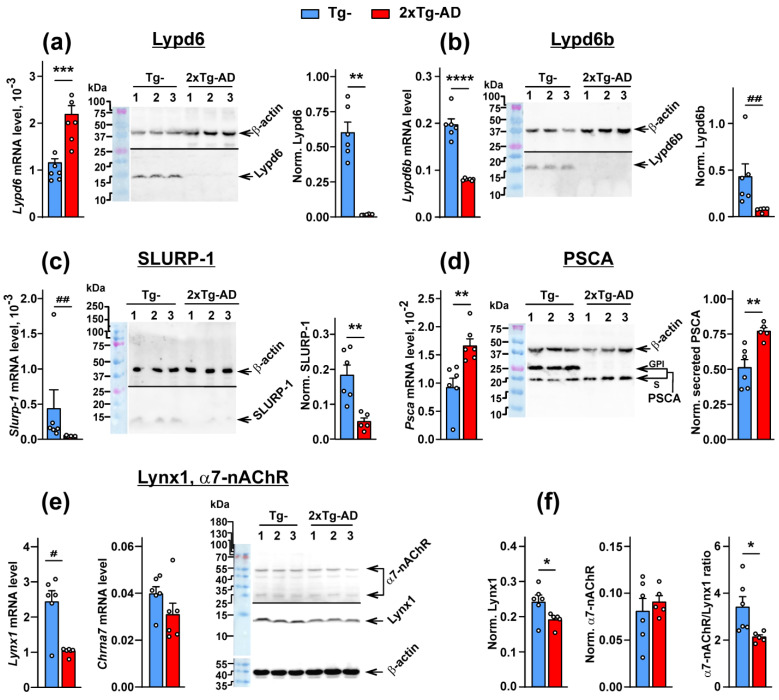
Analysis of changes in Lypd6, Lypd6b, SLURP-1, PSCA, Lynx1, and α7-nAChR expression in the cerebellum of Tg^−^ and 2Tg-AD mice. (**a**–**d**, **left panels**) Analysis of *Lypd6*, *Lypd6b*, *Slurp-1*, and *Psca* mRNA expression level normalized to expression level of the *β-actin*, *Gpdh*, and *Rpl13a* housekeeping genes and presented as relative mRNA level ± SEM (*n* = 6). (**a**–**d**, **central panels**) Representative Western blotting membranes for analysis of Lypd6, Lypd6b, SLURP-1, and PSCA expression in the cerebellum of three (1–3) Tg^−^ and three (1–3) 2xTg-AD mice. The positions of GPI-anchored and secreted PSCA are indicated as “GPI” and “S”, respectively. The whole Western blotting membranes are shown in Figure S1a–d. (**a**–**d**, **right panels**) Quantification of protein expression level of Lypd6, Lypd6b, SLURP-1, and PSCA normalized to β-actin expression level, the data presented as normalized protein band intensity ± SEM (*n* = 6 for Tg^−^ and 5 for 2xTg-AD mice). (**e**) Analysis of Lynx1 and α7-nAChR expression. Gene expression level of *Lynx1* and *Chrna7* (**left panel**) was normalized to expression level of the *β-actin*, *Gpdh,* and *Rpl13a* housekeeping genes and presented as relative mRNA level ± SEM (*n* = 6). Representative Western blotting membrane for analysis of α7-nAChR and Lynx1 expression in the cerebellum of three (1–3) Tg^−^ and three (1–3) 2xTg-AD mice is shown at the right panel. The whole Western blotting membranes are shown in Figure S1e. (**f**) Analysis of expression of the Lynx1 and α7-nAChR proteins normalized to β-actin expression, the data presented as normalized protein band intensity ± SEM (*n* = 6 for Tg^−^ and 5 for 2xTg-AD mice). On the right panel, analysis of the α7-nAChR/Lynx1 ratio is presented (*n* = 6 for Tg^−^ and 5 for 2xTg-AD mice). * (*p* < 0.05), ** (*p* < 0.01), *** (*p* < 0.001), and **** (*p* < 0.0001) indicate significant difference between the data groups according to the two-sided *t*-test. # (*p* < 0.05) and ## (*p* < 0.01) indicate significant difference between the data groups according to the Mann–Whitney u-test.

Upon Aβ accumulation, the changes in expression of the genes associated with Aβ clearance, synaptic and neuronal plasticity, and neuroprotection are observed in the brain [31,32,33]. Here, we detected down-regulated expression of the genes coding the SLURP-1, Lypd6b and Lynx1 proteins in the cerebellum of 2xTg-AD mice in comparison with non-transgenic (Tg^−^) mice (Figure 1b,c,e and Figure S1a,d,e). SLURP-1, Lypd6b, and Lynx1 expression was also diminished at the protein level (Figure 1b,c,e). Surprisingly, the mRNA level of *PSCA* and *Lypd6* was up-regulated in 2xTg-AD mice, while expression of GPI-anchored forms of the PSCA and Lypd6 proteins was fully absent in the cerebellum of 2xTg-AD mice in comparison with Tg^−^ mice (Figure 1a,d). Notably, PSCA can be expressed both in the membrane tethered by GPI-anchor and secreted (without GPI-anchor) forms [22,34]. Here, we revealed two forms of PSCA in the cerebellum of Tg^−^ mice and only the secreted form in 2xTg-AD mice, and the amount of secreted PSCA was significantly higher in transgenic mice (Figure 1d and Figure S1c). 

No changes in the *Lynx2* and *Slurp-2* mRNA expression were detected (Figure S2a,b). Expression of α7-nAChR did not change either at the mRNA or protein levels (Figure 1e,f).

### 2.2. Colocalization of Lynx1 with α7-nAChR Is Deacreased in the Cerebellum of 2xTg-AD Mice

**Figure 2:**
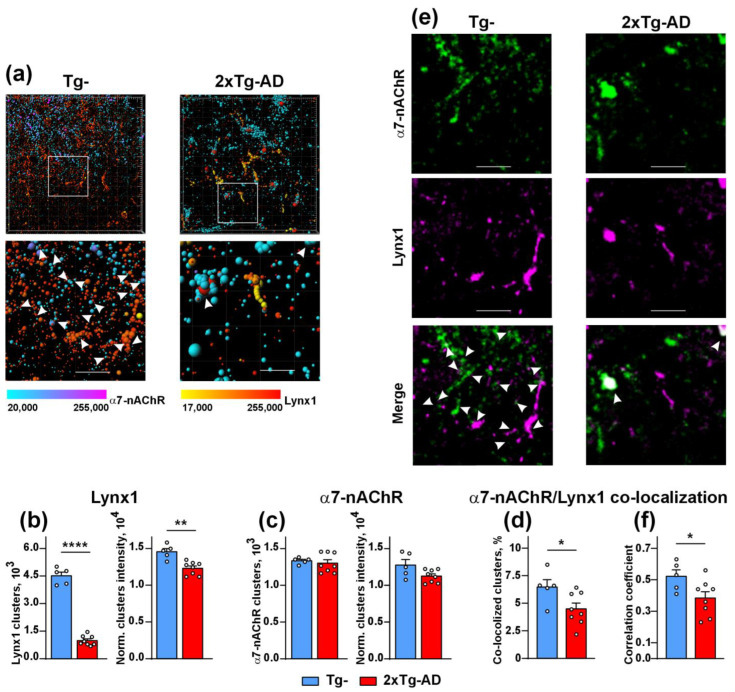
Analysis of Lynx1 and α7-nAChR expression and their co-localization in the cerebellum of Tg^−^ (*n* = 5) and 2Tg-AD (*n* = 8) mice. (**a**) Examples of the Lynx1 and α7-nAChR clusters reconstructed using the Imaris software. The contacting Lynx1 and α7-nAChR clusters are indicated by the arrows, scale—10 µm. (**b**,**c**) Analysis of the quantity and intensity (left and right panels, respectively) of the Lynx1 and α7-nAChR clusters. The data are presented as the number of the clusters ± SEM or as the cluster staining intensity normalized to the intensity of background of image ± SEM. (**d**) % of the Lynx1 clusters co-localized with the α7-nAChR clusters. (**e**) Examples of the maximum intensity of z-stack projections in the slices of the cerebellum, the contacts between the Lynx1 (magenta) and α7-nAChR (green) clusters are indicated by the arrows, scale—10 µm. (**f**) Analysis of the Lynx1 and α7-nAChR clusters’ co-localization by the Pearson’s regression, the data presented as the correlation coefficient ± SEM. * (*p* < 0.05), ** (*p* < 0.01), and **** (*p* < 0.0001) indicate significant difference between the data groups according to the two-sided *t*-test.

From here, we concentrated our attention on Lynx1, as the most characterized endogenous neuromodulator responsible for the modulation of α7-nAChR and cognitive function in the brain. Co-localization of Lynx1 with α7-nAChR in the cortical, hippocampal, and amygdala neurons [26,28] and in the brains of Tg^−^ mice [26,28] has been reported. Here, we studied co-localization of Lynx1 with α7-nAChR in the cerebellum of 2xTg-AD and Tg^−^ mice. We found that both Lynx1 and α7-nAChR were expressed as clusters and were not distributed uniformly in the cerebellum. The clusters with volumes more than ~0.1 µm^3^ were reconstructed using the Imaris 8.0 software, and the dramatic reduction of the number of the Lynx1 clusters was revealed in the cerebellum of 2xTg-AD mice in comparison with non-transgenic mice (Figure 2a,b). The staining intensity of the Lynx1 clusters was also reduced, while the number and intensity of the α7-nAChR clusters was the same in 2xTg-AD and Tg^−^ mice (Figure 2a,c). Moreover, the number of the Lynx1 clusters co-localized with the α7-nAChR clusters was significantly lower in 2xTg-AD mice in comparison to Tg^−^ ones (Figure 2d,e), that was confirmed by the Pearson’s regression analysis (Figure 2f).

**Figure 3:**
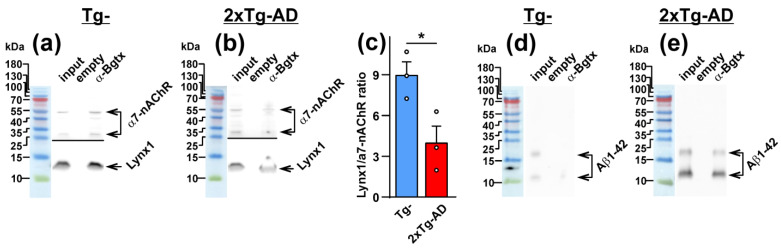
Analysis of the α7-nAChR partners extracted from the homogenate of cerebellum of Tg^−^ (**a**,**d**) and 2xTg-AD (**b**,**e**) mice by affinity extraction using NHS-Sepharose resin coupled with α-Bgtx (*n* = 3). The empty resin blocked by 500 mM ethanolamine +5% skim milk was used as a negative control. Analysis was performed by Western blotting. Whole Western blotting membranes are shown in Figure S3 for Tg^−^ mice and in Figure S4 for 2xTg-AD mice. For α7-nAChR, two bands were observed, corresponding to the whole and hydrolyzed subunits (shown by the arrows). The total intensity of the two bands was used for the quantification. (**c**) Analysis of the ratio of the intensities of the bands from (**a**,**b**) corresponding to the Lynx1 and α7-nAChR proteins extracted by α-Bgtx. Data presented as the Lynx1/α7-nAChR band intensity ratio + SEM (*n* = 3); * (*p* < 0.05) indicates significant difference between the data groups according to the two-sided *t*-test.

Previously, we showed the direct association of Lynx1 with α7-nAChR in the brain of wild-type mice [17]. Here, we performed the extraction of α7-nAChR/Lynx1 complexes from the cerebellum of Tg^−^ and 2xTg-AD mice using α-Bgtx (specific α7-nAChR antagonist) as a bait. Notably, α-Bgtx does not compete with Lynx1 for the binding with this receptor [35]; thus, the toxin does not prevent Lynx1 from interacting with α7-nAChR. α-Bgtx extracted a much lesser amount of Lynx1 from the homogenate of the cerebellum of 2xTg-AD mice compared with Tg^−^ mice (Figure 3a,b). The ratio of the amount of extracted Lynx1 to the amount of extracted α7-nAChR was significantly lower in 2xTg-AD mice (Figure 3c). This is in line both with reduced Lynx1 expression (Figure 1e,f) and diminished co-localization of this neuromodulator with α7-nAChR in the cerebellum (Figure 2d–f).

### 2.3. Increased Co-Localization and Direct Interaction of Aβ1-42 with α7-nAChR in the Cerebellum of 2xTg-AD Mice

**Figure 4:**
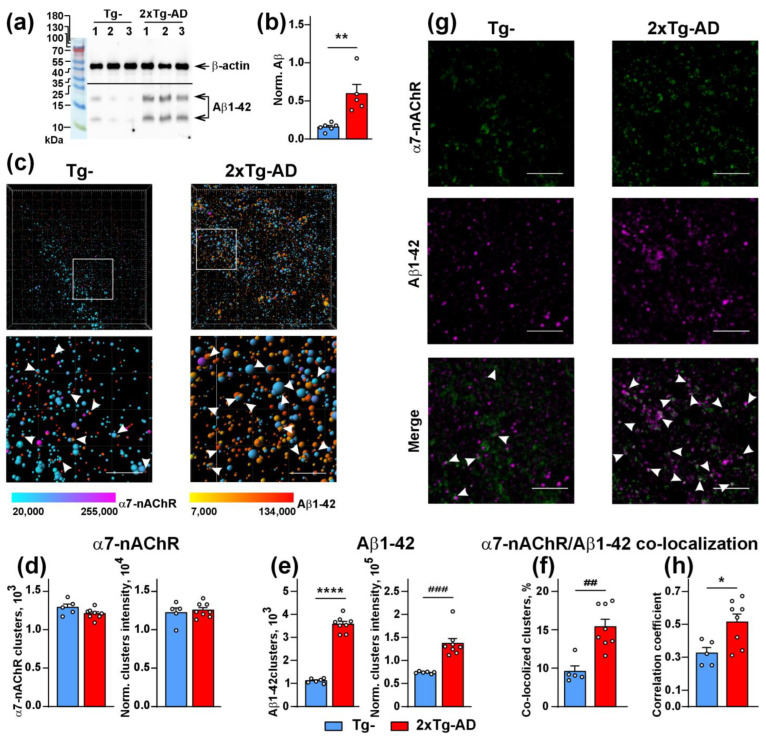
Analysis of α7-nAChR and Aβ1-42 expression and co-localization in the cerebellum of Tg^−^ and 2Tg-AD mice. (**a**) Example of analysis of Aβ1-42 expression in the cerebellum of three Tg^−^ and three 2xTg-AD mice by Western blotting. The whole Western blotting membranes are shown in Figure S5. (**b**) Analysis of expression level of Aβ1-42 normalized to expression level of β-actin; the data are presented as normalized protein band intensity ± SEM (*n* = 6 for Tg^−^ and 5 for 2xTg-AD mice). (**c**) Examples of the Aβ1-42 and α7-nAChR clusters reconstructed using the Imaris software, some contacting Aβ1-42 and α7-nAChR clusters are indicated by arrows, scale—10 µm. (**d**,**e**) Analysis of the quantity and intensity of the Aβ1-42 and α7-nAChR clusters. The data are presented as the number of the clusters ± SEM or as the cluster staining intensity normalized to the intensity of background of image ± SEM (*n* = 5 for Tg^−^ and 8 for 2xTg-AD mice). (**f**) % of the Aβ1-42 clusters co-localized with the α7-nAChR clusters in the cerebellum of Tg^−^ and 2xTg-AD mice (*n* = 5 for Tg^−^ and 8 for 2xTg-AD mice). (**g**) Examples of the maximum intensity of z-stack projections in the slices of the cerebellum of Tg^−^ and 2xTg-AD mice; the contacts between the Aβ1-42 (magenta) and α7-nAChR (green) clusters are indicated by the arrows, scale—10 µm. (**h**) Analysis of the Aβ1-42 and α7-nAChR clusters co-localization by the Pearson’s regression; the data are presented as the correlation coefficient ± SEM (*n* = 5 for Tg^−^ and 8 for 2xTg-AD mice). * (*p* < 0.05), ** (*p* < 0.01), and **** (*p* < 0.0001) indicate significant difference between the data groups according to the two-sided *t*-test ## (*p* < 0.01) and ### (*p* < 0.001) indicates a significant difference between the data groups according to the Mann-Whitney u-test.

APP accumulates in the cerebellum of 2xTg-AD mice from the age of 6 months [36]. Here, we investigated the expression and localization of Aβ1-42 in the cerebellum of 8-9-month-old Tg^−^ and 2xTg-AD mice. Analysis by Western blotting showed significantly increased levels of Aβ1-42 in 2xTg-AD mice in comparison with Tg^−^ mice (Figure 4a,b). Similarly to Lynx1 and α7-nAChR, Aβ1-42 formed the clusters in the cerebellum of both types of mice, but, as was expected, the cluster quantity and intensity was significantly higher in 2xTg-AD mice. These Aβ1-42 clusters had a minimum diameter of ~ 0.3 µm and a maximum diameter of several µm that agrees well with the previous study of amyloid plaques [36]. Most likely, the small Aβ1-42 clusters are the initial points for subsequent Aβ1-42 oligomerization. We also saw an increased number of the Aβ1-42 clusters contacting the α7-nAChR clusters (Figure 4c–f). Analysis of Pearson’s correlation coefficient confirmed the enhancement of co-localization of Aβ1-42 with α7-nAChR in the cerebellum of 2xTg-AD mice (Figure 4g,h). Moreover, direct interaction of Aβ1-42 with α7-nAChR in the cerebellum of 2xTg-AD mice, but not of Tg^−^ mice, was revealed by affinity extraction of the receptor by α-Bgtx (Figure 3d,e). Diminished expression of Lynx1 and its co-localization with α7-nAChR, together with increased co-localization of Aβ1-42 and its interaction with α7-nAChR, could result in dysregulation of this receptor in the cerebellum of 2xTg-AD mice.

### 2.4. Dimished Expression of KLF4 Transcription Factor, Lynx1 and SLURP-1 Together with α7-nAChR Dysfunction Results in Activation of Inflammation in the Cerebellum of 2xTg-AD Mice

**Figure 5:**
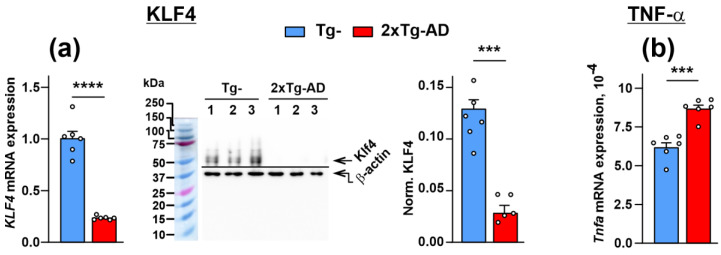
Analysis of KLF4 and TNF-α expression in the cerebellum of Tg- and 2xTg-AD mice. (**a**) Analysis of *Klf4* gene expression (**left**) and KLF4 expression at the protein level (**right**) in the cerebellum of Tg^−^ and 2xTg-AD mice. Gene expression was normalized to expression of *β-actin*, *Gpdh*, and *Rpl13a* housekeeping genes and presented as a relative mRNA level ± SEM (*n* = 6). Analysis of KLF4 protein expression was performed by Western blotting, and its quantification (*n* = 5–6) is shown on the right panel. The whole Western blotting membranes are shown in Figure S6. (**b**) *Tnfa* mRNA expression level was normalized to the expression levels of *β-actin*, *Gpdh*, and *Rpl13a* housekeeping genes and presented as the log of the relative mRNA level ± SEM (*n* = 6). *** (*p* < 0.001) and **** (*p* < 0.0001) indicate significant difference between the data groups according to the two-sided *t*-test.

Potentiation of α7-nAChR leads to the control of TNF-α release and inflammation in epithelial cells [37,38]. On the other hand, KLF4, an anti-inflammatory transcription factor [39], positively regulates expression of the *Lynx1* and *Slurp1* genes [40,41]. We analyzed the influence of the Aβ hyperexpression in the cerebellum of 2xTg-AD mice on *Klf4* expression using real-time PCR and revealed, similarly to *Lynx1* and *Slurp1*, significant down-regulation of this gene expression (Figure 5a). Western blotting confirmed the down-regulation of KLF4 at the protein level (Figure 5a). In line with decreased expressions of KLF4, Lynx1, and SLURP-1, and the proposed dysregulation of α7-nAChR, the expression of mRNA coding TNF-α was overexpressed in the cerebellum of 2xTg-AD mice (Figure 5b). This is in line with our previous observation of the increased level of TNF-α in the blood serum of 2xTg-AD mice [28].

### 2.5. Amyloidosis Promotes the Astrocyte Degeneration in the Cerebellum

**Figure 6:**
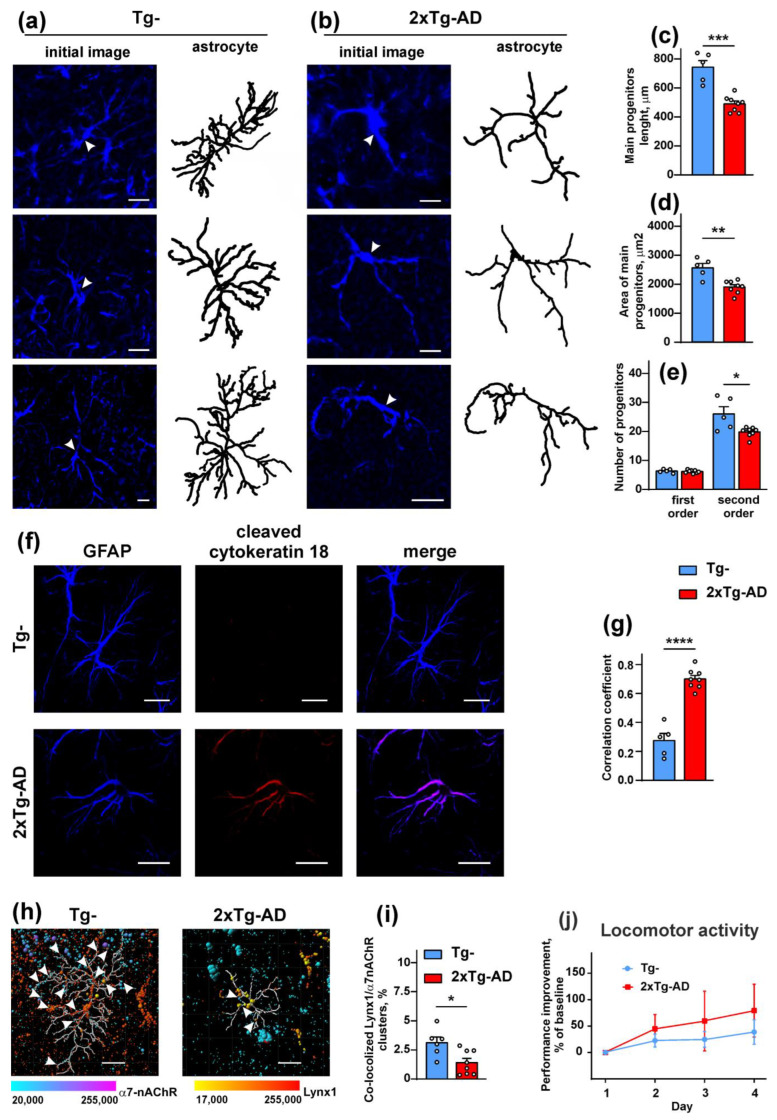
Analysis of astrocyte morphology, main progenitors’ area, and astrocyte branching in Tg^−^ and 2xTG-AD mice. (**a**,**b**) Representative images of astrocytes in the cerebellum of non-transgenic and transgenic mice, initial images, and reconstructed astrocyte morphology by the “filament tracer” of the Imaris software are shown at the left and right panels, respectively. Arrows show the positions of individual cells, scale—10 µm. (**c**) Average length of the main progenitors of individual astrocytes stained by GFAP was assayed using the Imaris software. (**d**) Area occupied by the astrocyte was calculated by the ImageJ 1.5 software. Please note that this area is not the actual volume of the astrocytic domain and indicates only the spread of the main progenitors of the astrocyte, which can be stained by a GFAP antibody. (**e**) Astrocytic progenitors of the first and second order were counted manually using the reconstructed main astrocytic progenitors. All data presented as mean ± SEM (*n* = 5 for Tg^−^ and 8 for 2Tg-AD mice); (**f**) Examples of the maximum intensity of z-stack projections in the slices of the cerebellum of Tg^−^ and 2xTg-AD mice, stained for cleaved cytokeratin 18 (magenta) and GFAP (blue), scale—10 µm; (**g**) Analysis of co-localization of the astrocyte stained by Ab to GFAP and cleaved cytokeratin 18; (**h**) Example of the Lynx1 and α7-nAChR clusters’ distribution within the astrocytic area. Arrows indicate the contacting Lynx1 and α7-nAChR clusters (**i**) Analysis of co-localization of the Lynx1 and α7-nAChR clusters within the astrocyte (*n* = 5 for Tg^−^ and 8 for 2xTg-AD mice). * (*p* < 0.05), ** (*p* < 0.01), *** (*p* < 0.001), **** (*p* < 0.0001) indicate a significant difference between the data groups according to the two-sided *t*-test. (**j**). Motor learning test in Tg^−^ (*n* = 13) and 2xTg-AD (*n* = 9) mice. The performance dynamics in the rotarod test over the 4 consecutive days is expressed as percentage of baseline (1-day) values.

Astrocytic and microglia α7-nAChRs contribute to Aβ metabolism, including Aβ phagocytosis and degradation, and to regulation of neuroinflammation [42]. At the same time, degeneration of astrocytes could be observed without detectable changes in neuronal and synaptic plasticity in early AD onset [43]. Here, we evaluated whether the Aβ pathology and α7-nAChR dysfunction can influence the astrocytes in the cerebellum of 2xTg-AD mice. Confocal microscopy revealed that the major GFAP-positive astrocytic progenitor’s length and the number of astrocytic progenitors of the secondary order were decreased in 2xTg-AD mice more than 1.6 fold in comparison with Tg^−^ mice (Figure 6a–c,e). No signs of astrocytic hypertrophy in the cerebellum of 2xTg-AD mice (increase in the area occupied by the main astrocytic progenitors) were observed (Figure 6a,b,d).

A decrease in the main astrocytic progenitor length led to a reduction of the area between the main astrocytic branches, however this parameter does not reflect an actual area of the astrocytic domain, which usually includes thin progenitors and astrocytic leaflets, which could not be stained by GFAP antibody [44] (Figure 6b).

Analysis of cleaved cytokeratin 18, which is usually cleaved by caspases and is a marker of apoptosis or necrosis [45], showed up-regulation of astrocytes with cleaved cytokeratin 18 in 2xTg-AD mice, pointing to the death of cerebellar astrocytes within Aβ pathology (Figure 6f,g). Analysis of co-localization of the Lynx1 and α7-nAChR clusters within the astrocyte revealed the reduced number of the co-localized Lynx1/α7-nAChR clusters in the cerebellum of 2xTg-AD mice (Figure 6f,h,i). Thus, Aβ hyperexpression leads to degeneration of the astrocytes and uncoupling of Lynx1 from α7-nAChR within the astrocytic progenitors in the cerebellum of 2xTg-AD mice.

To test if the changes in astrocytic morphology in the cerebellum of 2xTg-AD mice reflects impaired cognitive function, we performed the rotarod test usually used to study the locomotor activity associated with the cerebellum function. No changes in the locomotor performance were revealed of 2xTg-AD mice in comparison with Tg^−^ mice over a 4-day trial session (Figure 6j).

## 3. Discussion

Cerebellar dysfunction was observed in neurodegeneration and AD [12,46]. Aβ accumulation (but not in the form of actual senile plaques) in the cerebellum is often observed for early onset of AD before cognitive decline [12,47]. Aβ burden and cerebellar synaptic plasticity disruption were shown in mouse models of AD [36,48]. Besides amyloidosis, cholinergic system dysfunction is also characteristic for AD progression [49,50]; however, the implication of the cholinergic system in AD pathology in the cerebellum has not been previously studied. Here, we studied the implication of the endogenous modulators of the cholinergic system from the Ly6/uPAR family on cerebellum function upon early AD onset using the model based on transgenic mice with mutant APP and presenilin-1 overexpression (2xTg-AD).

The Ly6/uPAR family contains 38 human proteins, and 10 of them are modulators of the different nAChRs. Using qPCR and Western blotting, we demonstrated the reduction of expression of the modulators of α7-nAChR, Lynx1 [17,19] and SLURP-1 [17,19], in the cerebellum of 2xTg-AD mice (Figure 1c,e). At the same time, dramatically decreased expression of the GPI-anchored proteins Lypd6b (specific modulator of α3β4-nAChR [51]), Lypd6 (specific inhibitor of α7- and α3β4-nAChRs [15], and PSCA (targets nAChRs containing the α4 subunit, but not α7 one [27]) was revealed in the cerebellum of mice with Aβ amyloidosis (Figure 1a,b,d). These data suggest that not only α7-nAChR, but other types of nicotinic receptor can be implicated in AD onset.

Previous studies revealed non-changed Lypd6 protein expression and increased expression of secreted PSCA in the medial frontal gyrus of AD patients [30]. In line with that data, the secreted form of PSCA also was found to be significantly increased in the cerebellum of 2xTg-AD mice (Figure 1d). In contrast, no Lypd6 and PSCA expression in the form of GPI-anchored proteins was detected in the cerebellum of 2xTg-AD mice (Figure 1a,d). This difference can be explained either by non-uniform expression of nAChRs and their modulators in the different brain areas and different types of neurons [52,53,54,55], or by a disrupted mechanism of the GPI-anchoring of the Ly6/uPAR proteins to the cell membrane. The latter assumption is supported by the fact that mRNA expression of *Lypd6* and *Psca* was increased in the cerebellum of 2xTg-AD mice without visible protein expression in the membrane-tethered form (Figure 1a,d). Increased mRNA expression in these cases could be an attempt of the cell to compensate for the diminished number of GPI-anchored proteins inside the cell. As a result, an increased amount of the secreted PSCA is observed outside the cell. Notably, the secreted form of Lypd6 was not reported presently.

**Figure 7:**
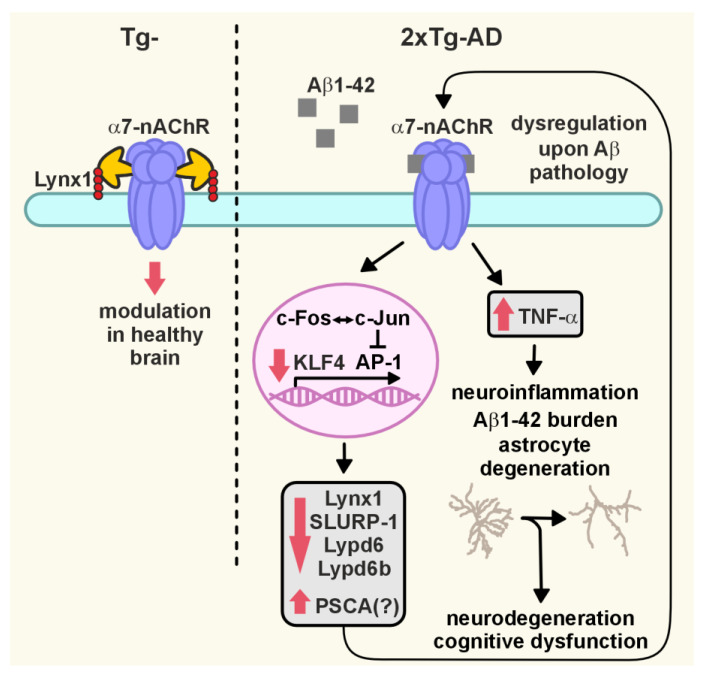
Suggested model of the cholinergic dysregulation in the cerebellum of 2Tg-AD mice. Aβ1-42 burden leads to down-regulation of expression of anti-inflammatory transcription factor KLF4, regulating expression of the Ly6/uPAR proteins, which in turn modulate α7-nAChR function. This drives interaction of Aβ1-42 with α7-nAChR, which results in dysregulation of this receptor, promotes neuroinflammation with increased expression of inflammatory cytokine TNF-α, and leads to degeneration of astrocytes and further neurodegeneration and cognitive function impairment.

The down-regulation of Lynx1 expression in the cortexes of 3xTg-AD mice was shown earlier [27]. Moreover, we showed previously that Aβ1-42 induces activation of JNK, which is usually accompanied by c-Jun activation [56] and c-Fos down-regulation [57]. The Lynx1 promoter can be activated by the AP-1 transcription complex, which consists both of c-Jun and c-Fos, so a possible imbalance between c-Jun and c-Fos expression can cause decreased formation of the AP-1 complex, which in turn, leads to *Lynx1* down-regulation with a consequent decrease in protein level. At the same time, the water-soluble domain of Lynx1 (ws-Lynx1) potentiates α7-nAChR in the cortex, stimulates synaptic plasticity in the hippocampus [17], competes with Aβ1-42 for the binding to α7-nAChR [27], and compensates the deficiency of endogenous Lynx1 expression and LTP impairment in the hippocampal neurons observed upon incubation with Aβ1-42 [27,58]. Thus, diminished Lynx1 expression observed here in the cerebellum of 2xTg-AD mice could crucially affect α7-nAChR and, as a result, cognitive function too. Here, we suggest the mechanistic model which could explain Lynx1 participation in pathogenesis of cerebellar amyloidosis and α7-nAChR dysfunction (Figure 7). In the normal brain, Lynx1 is tethered to the cell membrane by the GPI-anchor and is physically associated with α7-nAChR, as we showed earlier [17]. Within the onset of Aβ pathology, the expression of Lynx1 is decreased (Figure 1e,f); the Lynx1 amount associated with α7-nAChR in the cerebellum becomes lower (Figure 2d and Figure 3c) and insufficient to regulate the receptor’s function properly. The α7 receptor not “shielded” by Lynx1 becomes accessible for the interaction with toxic Aβ1-42 (Figure 4), which in turn leads to further dysfunction of the cholinergic system. In line with our hypothesis, the knock-out of the Lynx1 gene in mice results in nAChR-mediated degeneration in axonal tracts of the cerebellum and in dorsal striatum [59]. Notably, the roles of other nAChR modulators such as Lypd6 [15], Lypd6b [51], and SLURP-1 [17,19], as well as PSCA, in dysregulation of the cholinergic system and AD progression is not studied yet. Here, we can only propose their participation based on significantly changed expression (Figure 1a–d).

Inflammation in the brain is one of the characteristic features of AD [60,61,62]. At the same time, α7-nAChR plays an essential role in the control of differentiation and inflammation in epithelial cells, and particularly in the control of release of inflammation cytokine TNF-α [37,38,63]. In line with this, here we observed decreased expression of KLF4, the transcription factor regulating the differentiation of different cells, which possess anti-inflammatory activity in AD [39,64] (Figure 5a), with simultaneous increased expression of TNF-α mRNA (Figure 5b). At the same time, the *KLF4* knock-down can repress the *Lynx1* transcription in the corneal epithelium [41], while KLF4 overexpression drives expression of SLURP-1 in epithelial cells [40]. Thus, in addition to possible imbalance of the AP-1 complex formation, KLF4 down-regulation may be another reason for the Lynx1 down-regulation in Aβ pathology. Notably, increased TNF-α and decreased SLURP-1 amounts were recently reported in the blood serum of 2xTg-AD mice in comparison with non-transgenic mice [28]. Altogether, these data point to direct correlation between decreased expression of KLF4 and α7-nAChR modulators (Lynx1, SLURP-1), α7-nAChR dysfunction, and activation of inflammatory processes in the cerebellum upon Aβ accumulation (Figure 7).

The astrocytic degeneration in the hippocampus within AD progression was shown previously for 9-month-old 3xTg-AD mice with both Aβ and tau pathologies [65,66,67,68] and in the postmortem cortexes of AD patients [69]. Generally, it was proposed that astroglia degeneration may drive the cognitive abnormalities in early AD [65]. However, the influence of amyloidosis on the cerebellar astrocytes remained unclear [70]. Here, we revealed the degenerative changes in the astrocytic branching in the cerebellum within early AD onset (Figure 6a–e), accompanied by astrocytic death (Figure 6f,g), and the reduced number of the co-localized Lynx1 and α7-nAChR clusters in the vicinity of the astrocyte (Figure 6h,i). Together with inhibition of long-term potentiation in the cerebellum of 2xTg-AD mice [36,48], this may point to implication of the astrocytes in impaired cerebellar synaptic plasticity. However, no changes in the locomotor activity in the Rotarod test usually associated with cerebellum function were revealed (Figure 6j). Astrogliosis (astrocytic hypertrophy accompanied with metabolic changes) is another facet of astroglia changes in AD [65,68]. However, we did not observe astrocytic hypertrophy in the cerebellum (Figure 6a,d). Presumably, astrogliosis is manifested only in the later AD stages, when big Aβ plaques are formed. In line with this, astrocytic hypertrophy in the hippocampi of 3xTg-AD mice of 12-month age was characteristic only for astrocytes surrounding Aβ plaques [67]. Thus, in early AD onset, changes in expression of the Ly6/uPAR proteins, dysfunction of the cholinergic system, and astrocytic degeneration in the cerebellum have already taken place, while astrogliosis and cognitive dysfunction are not yet evident.

In conclusion, we described, for the first time, the changes in the cerebellum occurring upon Aβ accumulation in early AD onset. Changes in expression of the several Ly6/uPAR proteins followed by dysregulation of the cholinergic system, as well as degeneration of the cerebellar astrocytes were revealed without detectable changes in cognitive function. Compensation of dysregulation of α7-nAChR function may be a prospective strategy to prevent early AD onset or compensate for cognitive decline in AD.

## 4. Materials and Methods

### 4.1. Animals

B6C3-Tg(APP695)85Dbo Tg(PSEN1)85Dbo double transgenic (APP/PS1) adult mice expressing a chimeric mouse/human amyloid precursor protein with T714I mutation (Mo/HuAPP695swe) and a mutant of human presenilin-1 (PS1-dE9) designated as 2Tg-AD and non-transgenic littermates (designated as Tg^−^) were purchased from the Jackson laboratory (Bar-Harbor, ME, USA). Both APP/PS1 mutations are associated with early-onset AD.

Initially, the animals were bred and housed under the standard conditions of the Animal Breeding Facility, BIBCh, RAS (the Unique Research Unit Bio-Model of the IBCh, RAS; the Bioresource Collection—Collection of SPF-Laboratory Rodents for Fundamental, Biomedical and Pharmacological Studies), accredited at the international level by AAALACi, from which genotyped 6-month-old animals (initially 13 Tg^−^ and 14 2xTg-AD) were purchased for the experiments. Animals were kept in the SPF zone of vivarium of IBCh RAS until 9 months of age (30–35 g at the end of experiments). Experiments were carried out on 9-month-old mice. The Tg^−^ group consisted of 6 males and 7 females; the 2xTg-AD group consisted of 7 males and 7 females. After the rotarod test, the animals were euthanized using a CO_2_ chamber (Acrylmedic, Romashkovo, Russia); after that, cerebellum was isolated and stored in liquid nitrogen (6 Tg^−^ and 6 2xTg-AD mice) for Western blotting, PCR, or affinity purification, or fixed in 4% paraformaldehyde (Panreac, Barcelona, Spain) for immunohistochemistry (5 Tg^−^ and 8 2xTg-AD mice). All analyses were performed at the same time point.

All procedures were performed in accordance with Rus-LASA ethical recommendations approved by the Institute of Bioorganic Chemistry ethical committee (protocol #318/2021).

### 4.2. Real-Time PCR for mRNA Detection

Total RNA from the homogenate of the cerebellum was isolated using the Bio-Rad Aurum RNA mini-isolation kit (Bio-Rad, Hercules, CA, USA) according to the manufacturer’s instructions. cDNA was synthesized from 2 µg of total RNA by the Mint reverse transcriptase kit and oligodT primer (Evrogen, Moscow, Russia). After that, qPCR was performed with ready-to-use SYBR Green HS mix (Evrogen), 200 ng of cDNA, and the primers specific to the mouse *Lynx1*, *Lynx2*, *Lypd6*, *Lypd6b*, *Psca*, *Slurp1*, *Slurp2*, *Chrna7*, *Tnfa*, and *Klf4* genes (Table S1); these coded Lynx1, Lynx2, Lypd6, Lypd6b, prostate-specific cell antigen PSCA, SLURP-1, SLURP-2, α7-nAChR, Tnfα, and KLF4 proteins, respecively. Negative controls containing all the components of the PCR mixture with cDNA replaced by mRNA gave no signal. PCR reactions were carried out using the Roche LightCycler 96 amplifier (Roche, Basel, Switzerland). The mRNA expression level was normalized to the *β-actin*, *Gpdh*, and *RPL13a* housekeeping genes using the LightCycler SW 1.0 software (Roche).

### 4.3. Western Blotting and Affinity Purification

The cerebellum was homogenized in RIPA buffer containing Sifmafast protease inhibitor cocktail (S8820, Sigma-Aldrich, St Louis, MS, USA), lyzed for 30 min in ice, and sedimented at 16,000 g; the total protein concentration was measured by the BCA assay (Sigma-Aldrich), and probes were resuspended in the PAGE loading buffer. The probes were submitted to gel electrophoresis (10 µg of the total protein per lane), blotted onto nitrocellulose membranes (Bio-Rad), and blocked for 2 h in 5% skim milk (Sigma-Aldrich) in the TBS buffer with 0.1% Tween-20 (Applichem, Darmstadt, Germany). The membranes were incubated overnight at 4 °C with the primary antibody against Lypd6 (rabbit, 1:1000, ABIN5582866, Antibodies-online, Aachen, Germany), Lypd6b (rabbit, 1:1000, Abcam, Cambridge, UK), SLURP-1 (rabbit, ab93840, Abcam), PSCA (rabbit, 1:1000, ABIN3186575, Antibodies-online), α7-nAChR (rabbit, 1:1000, ab10096, Abcam, Cambridge, UK), Lynx1 (rabbit, 1:1000, ab12035, Abcam), and Aβ1-42 (rabbit, 1:2000, 12843, Cell Signaling, Danvers, MA, USA), washed 3 times with TBS + 0.1% Tween-20 and incubated with the HRP-conjugated secondary anti-rabbit antibody (1:5000, 111-035-003, Jackson Immunoresearch, West Grove, PA, USA) for 1 h at 20 °C. After that, membranes were washed 4 times with TBS + 0.1% Tween-20, and the HRP signal was detected by the ECL substrate (Bio-Rad) using the ImageQuant LAS 500 chemidocumenter (GE Healthcare, Chicago, IL, USA). For protein normalization, the same membrane was incubated for 1 h with anti-β-actin antibodies (mouse, 1:2000, mab8929, R&D Systems, Minneapolis, MN, USA), washed 3 times with TBS + 0.1% Tween-20, and incubated with the anti-mouse antibody (1:5000, 715-035-150, Jackson Immunoresearch) for 1 h at 20 °C. After that, membranes were washed 4 times with TBS + 0.1% Tween-20, and the HRP signal was detected by the ECL substrate (Bio-Rad, USA) using the ImageQuant LAS 500 chemidocumenter (GE Healthcare).

For investigation of the molecular partners of α7-nAChR in the cerebellum of Tg^−^ and 2xTg-AD mice, the α7-specific ligand—α-Bungarotoxin (α-Bgtx, 1 mg/mL, Tocris, Bristol, UK)—was coupled to NHS-activated Sepharose 4 Fast Flow (Cat #17-0906-01, GE Healthcare) according to the manufacturer’s instructions. The resin was blocked by 1% skim milk with 500 mM ethanolamine without any protein coupled and was used as a negative control (empty resin). The membrane fraction of the cerebellum (~0.005 mg per mouse) was solubilized in 2% Triton X-100 (Cat# A4975, Panreac), diluted 10 times with TBS, and incubated with the resin for 16 h at 4 °C. After that, non-specifically bound proteins were sequentially washed out from the resin with five volumes of TBS, five volumes of TBS + 1 M NaCl + 0.5% Triton X-100, and five volumes of TBS + 0.5% Triton X-100. The specifically bound proteins were eluted by five volumes of 200 mM glycine (pH 2.6) and diluted in the loading buffer (120 mM Tris–HCl, 20% [*v*/*v*] glycerol, 10% [*v*/*v*] mercaptoethanol, 4% [*w*/*v*] sodium dodecyl sulfate, and 0.05% [*w*/*v*] bromophenol blue, pH 6.8). Probes were submitted to gel electrophoresis, blotted onto nitrocellulose membranes (Bio-Rad) and blocked for 2 h in 5% skim milk (Sigma-Aldrich) in TBS + 0.1% Tween-20 (Applichem). The membranes were then incubated overnight at 4 °C with primary antibodies against α7-nAChR (rabbit, 1:1000, ab10096, Abcam), Lynx1 (rabbit, ab12035, Abcam), and Aβ1-42 (rabbit, 1:2000, 12843, Cell Signaling), washed 3 times with TBS + 0.1% Tween-20, and incubated with HRP-conjugated secondary anti-rabbit antibody (1:5000, 111-035-003, Jackson Immunoresearch). After that, membranes were washed 4 times with TBS + 0.1% Tween-20, and an HRP signal was detected by ECL substrate (Bio-Rad) using the ImageQuant LAS 500 chemidocumenter (GE Healthcare).

### 4.4. Immunohistochemistry

The cerebellum from Tg^−^ and 2Tg-AD mice was fixed for 16 h in 4% paraformaldehyde solution in the phosphate-buffered saline buffer (PBS). After that, the fixed cerebellum was incubated for 24 h in a 15% sucrose solution in PBS and for 24 h in a 30% sucrose solution in PBS. Then the cerebellum was dissected coronally by cryotome (M3-2, Kharkiv, Ukraine) on 25-μm thick slices. The middle cerebellar zone (25% of the cerebellum’s length forward from the caudal margin) was taken for immunohistochemistry. One slice from each mouse was analyzed. Heat epitope retrieval was performed in Declere buffer (ESBE Scientific, Markham, ON, Canada) for 20 min at 90 °C.

For the astrocyte reconstruction and α7-nAChR and Lynx1 staining, slices were incubated for 72 h with primary goat anti-GFAP (St John’s Laboratory, stj71028, 1:200, London, UK), mouse anti-α7-nAChR (Thermo Fisher, MA5-31691, 1:150, Waltham, MA, USA), and rabbit anti-Lynx1 (Abcam, ab125035, 1:200, Cambridge, UK) antibodies. For staining of α7-nAchR and Aβ1-42, the slices were stained for 72 h with mouse anti-α7-nAChR (Thermo Fisher, MA5-31691, 1:150) and rabbit anti-Aβ1-42 (Cell Signaling, 12843, 1:200, Danvers, MA, USA) antibodies. For the analysis of cleaved cytokeratin 18 localization within astrocytes, slices were incubated for 72 h with primary mouse anti-cleaved cytokeratin 18 antibodies (Primebiomed, 10-310011-01, 1:200, Moscow, Russia). Then slices were washed three times in Earle’s balanced salt solution (EBSS) and incubated for 2 h with donkey anti-goat Alexa405-conjugated (Abcam, ab175664, 1:500), donkey anti-mouse Alexa488-conjugated (Jackson Immunoresearch, 715-545-150, 1:500, West Grove, PA, USA), and chicken antirabbit Alexa647-conjugated (Life Technologies, A21443, 1:500, Waltham, MA, USA) secondary antibodies.

After washing three times in EBSS, slices were placed on polylysine-treated slides (Thermo Fisher, J2800AMNZ), embedded in Prolong Gold antifade mounting medium (Life Technologies), and the cerebellar cortex was observed under 63× (1.4) oil-immersion objective on Carl Zeiss LSM710 inverted confocal microscope for z-stack slice reconstructions. The square of the resulting images was 17,550 µm^2^.

### 4.5. Image Analysis

To reconstruct the astrocyte main progenitors stained by GFAP, we used the “filament tracer” tool of the Imaris 8.2. software (Imaris, Oxford, UK, soma diameter was set to 10 µm and thinnest progenitor to 0.17 µm). After automatic tracing, the reconstructed astrocyte was corrected with a visual inspection. After the astrocyte reconstruction, the length of all astrocytic progenitors was measured and the number of the progenitors of the first and second order were calculated manually. Also, the area of z-stack projection of the astrocyte was calculated using ImageJ and presented as the “astrocytic main progenitor area” in µm^2^. It should be noted that GFAP staining does not allow reconstruction of the whole astrocytic domain and it can roughly estimate the area of main astrocytic progenitor’s propagation. For each mouse, three cerebellum slices (12 astrocytes per mouse) were analyzed and data were averaged.

To quantify the expression of α7-nAChR, Lynx1, and Aβ1-42 demonstrating punctate distribution within the cerebellum slices, the “spot” instrument of Imaris was used. The clusters with minimum 0.3 µm diameter (corresponding to puncta volume ~ 0.1 µm^3^) were detected automatically. The cluster’s number and the intensity of the cluster’s fluorescence were determined automatically. The cluster’s stain intensity was normalized to background fluorescence. Examples of data analysis by Imaris are in Figure S7; the negative control slices, stained only by secondary antibodies are shown in Figure S8.

For co-localization analysis, the ImageJ software (NIH, Bethesda, MD, USA) was used. Each image of the z-stack was filtered using the unsharp mask (σ = 25), then images were thresholded (percentile, 90% + standard deviation of pixel intensities), and the co-localization of pixels in each z-stack plane was analyzed using the Coloc2 plugin of ImageJ. The negative control slices, stained only by secondary antibodies are shown in Figure S8. For each mouse, 3–4 fields of view from one cerebellum slice (the area of each view was ~X µm^2^) were analyzed and data were averaged.

### 4.6. Rotarod Motor Performance Test

The Rotarod test (Neirobotics, Zelenograd, Russia) was performed over four consecutive days. Tests were conducted at approximately the same time in the afternoon (1:00 to 8:00 p.m.). Throughout these days animals were allowed to habituate in the experimental room for at least 1 h before the tests. Mice (*n* = 9 for Tg^−^ and *n* = 13 for 2xTg-AD) were extensively handled for 3 days before the tests. Each session consisted of 5 trials. During a 3 min trial, the rotation was accelerated from 5 to 30 RPM and time spent by a mouse on the rod was recorded. The results of 5 trials were averaged and presented as time on accelerating roller normalized to the 1st day.

### 4.7. Statistical Analysis

Data are presented as mean ± SEM. Sample numbers (*n*) are indicated in the figure legends. The numbers of animals for Western blotting, affinity purification, and PCR were taken in accordance with previous study [17]. The number of sections for immunohistochemistry was selected based on the quality of the sections and on the number of cells that could be adequately reconstructed in the fields of view. No exclusion criteria were applied for the experimental data. Before the comparisons, the data were tested for normality (Shapiro-Wilk test, at *p* = 0.05). The data were analyzed using the two-tailed *t*-test for the normally distributed data, and a two-tailed Mann–Whitney u-test for the data with non-Gaussian distribution as indicated in the figure legends. Differences in the data were considered statistically significant at *p* < 0.05. Analysis was performed using the GraphPad Prism 8.0 software (GraphPad Software, San-Diego, CA, USA).

## Data Availability

Data available upon request.

## Supplementary Materials

The following supporting information can be downloaded at: https://www.mdpi.com/article/10.3390/ijms241914852/s1.
