# Supplementary material for: Aβ1-42 Accumulation Accompanies Changed Expression of Ly6/uPAR Proteins, Dysregulation of the Cholinergic System, and Degeneration of Astrocytes in the Cerebellum of Mouse Model of Early Alzheimer Disease
Source: Int J Mol Sci. 2023 Oct 3;24(19):14852. doi: 10.3390/ijms241914852 (PMC10573428; doi:10.3390/ijms241914852)
Supplement: Supplementary file 1 [file ijms-24-14852-s001.zip › ijms-2628940-supplementary.pdf]

Supplementary Information

Aβ1-42 accumulation accompanies changed expression of Ly6/uPAR proteins, dysregulation of the cholinergic system, and degeneration of astrocytes in the cerebellum of mouse model of early Alzheimer disease

Maxim L. Bychkov[1], Aizek B. Isaev[1,2], Alexander A. Andreev-Andrievsky[3,4], Konstantin Petrov[5], Alexander S. Paramonov[1], Mikhail P. Kirpichnikov[1,3], and Ekaterina N. Lyukmanova[6,1,2,3,*]

1 Shemyakin-Ovchinnikov Institute of Bioorganic Chemistry, Russian Academy of Sciences, 119997, Moscow, Russia.
2 Moscow Institute of Physics and Technology, State University, 141701 Dolgoprudny, Russia.
3 Interdisciplinary Scientific and Educational School of Moscow University «Molecular Technologies of the Living Systems and Synthetic Biology», Faculty of Biology, Lomonosov Moscow State University, 119234, Moscow, Russia
4 Institute for biomedical problems of Russian Academy of Sciences, 123007, Moscow, Russia
5 Arbuzov Institute of Organic and Physical Chemistry, Federal Research Center "Kazan Scientific Center of the Russian Academy of Sciences", Arbuzov str., 8, Kazan, 420088, Russian Federation
6 Shenzhen MSU-BIT University, 518172, Shenzhen, China
*Correspondence: lyukmanova_ekaterina@smbu.edu.cn (E.N.L.)

Table S1. Primers, used for qPCR experiments.

Gene	Primer		Amplicon size, bp
	Forward	Reverse	
β-actin	GCA GCC ACT GTC GAG TC	ACG ATG GAG GGG AAT ACA GC	88
Gpdh	GAG TTC CAG CGC AAG GGT TT	GGG GTC AGT CCA GTG CCA TA	165
Rpl13a	CGC TGT GAA GGC ATC AAC AT	TGG CCT CTC TTG GTC TTG TG	104
Tnfa	AGG CAC TCC CCC AAA AGA TG	GCT CCT CCA CTT GGT GGT TT	219
Klf4	TGC GGC AAA ACC TAC ACA AAG	GTT CAT CTG AGC GGG CGA AT	121
Lynx1	CAACACCGCACGAAGTGTG	GCCTGAGCTCTTGGTCTCT	101
Chrna7	TGCCACATTCCACACCAAC	TCCCATGAGATCCCATTCTC	142
Lynx2	GAG GAT GTG GGT TCT CGG C	CAG GGG ACG AGC AAT CGT T	125
Lypd6	CTG TCA CCA AAC GCT GTG TC	TTG TAG CCT TCG TGC TCT GAG	97
Lypd6b	ACT TGC GAA AAT GCA GGG GAT	GCT GGT GAA GTG ATG AAC TGT CA	76
Psca	TGC TGC TTG CCC TGTTGA T	CCT GTG AGT CAT CCA CGC A	138
Slurp1	AGG TCT CGG AAG CAG CAG AA	GGA CCA TTA CCC GCT GCA A	52
Slurp2	GAG GGA CTC CAC CCA CTG TGT	GCA GCC TAT GTG GCA CAT CTT	94

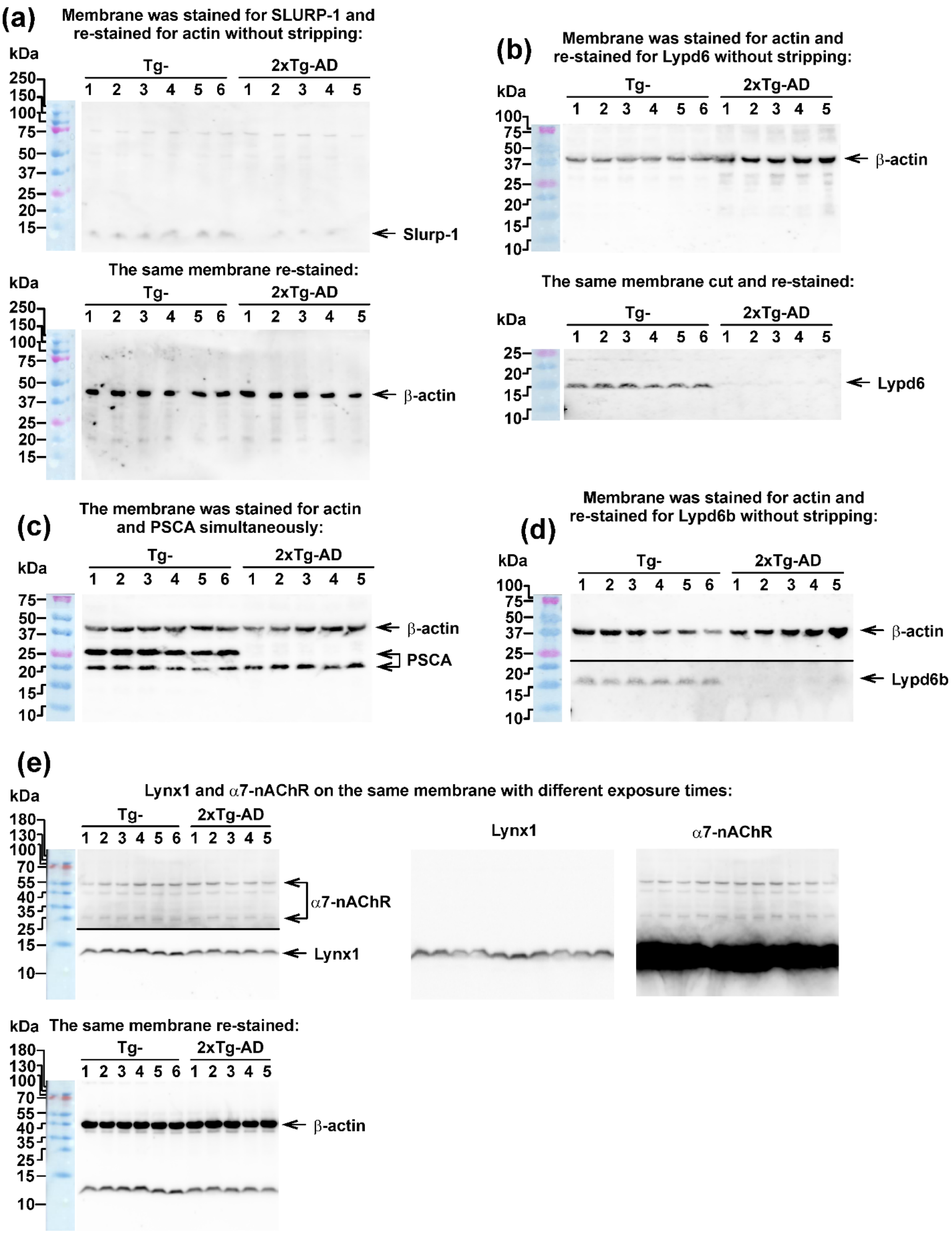

Figure S1. Whole Western blotting membranes showing the expression of SLURP-1 (a), Lypd6 (b), Lypd6b (c), PSCA (d), α7-nAChR, and Lynx1 (e) in the cerebellum of Tg- and 2xTg-AD mice.

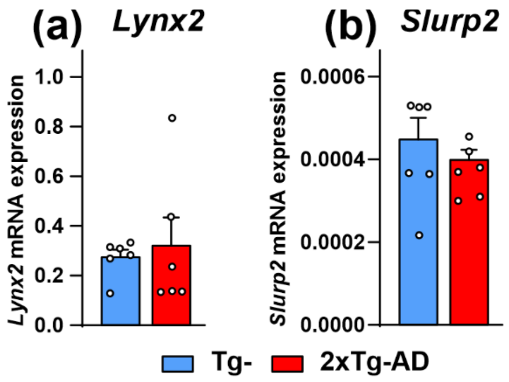

Figure S2. qPCR analysis of the expression of *Lynx2*, and *Slurp2* genes in the cerebellum of Tg$^-$ and 2xTg-AD mice. Gene expression was normalized to the *β-actin*, *Gpdh*, and *RPL13a* housekeeping genes and presented as relative mRNA level ± SEM (n = 6).

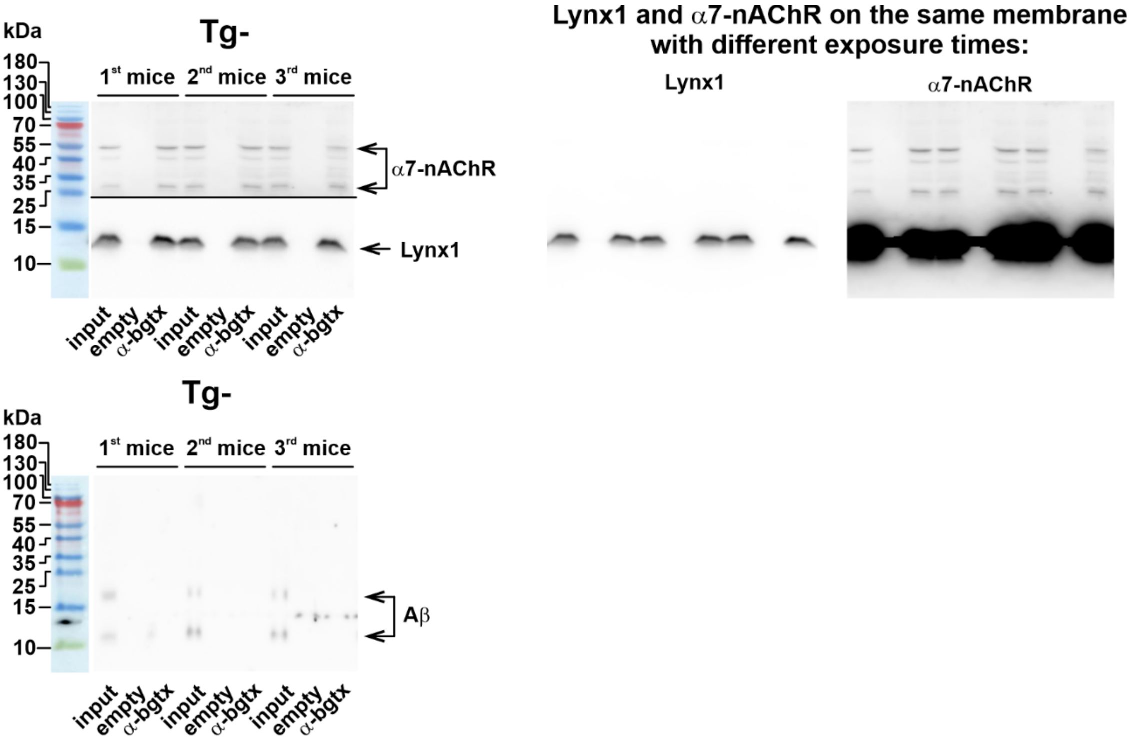

Figure S3. The whole Western-blotting membranes showing the α7-nAChR partners in the cerebellum of Tg$^-$ mice by affinity extraction using NHS-Sepharose resin coupled with α-Bgtx (n = 3). The empty resin blocked by 500 mM ethanolamine +5% skim milk was used as a negative control.

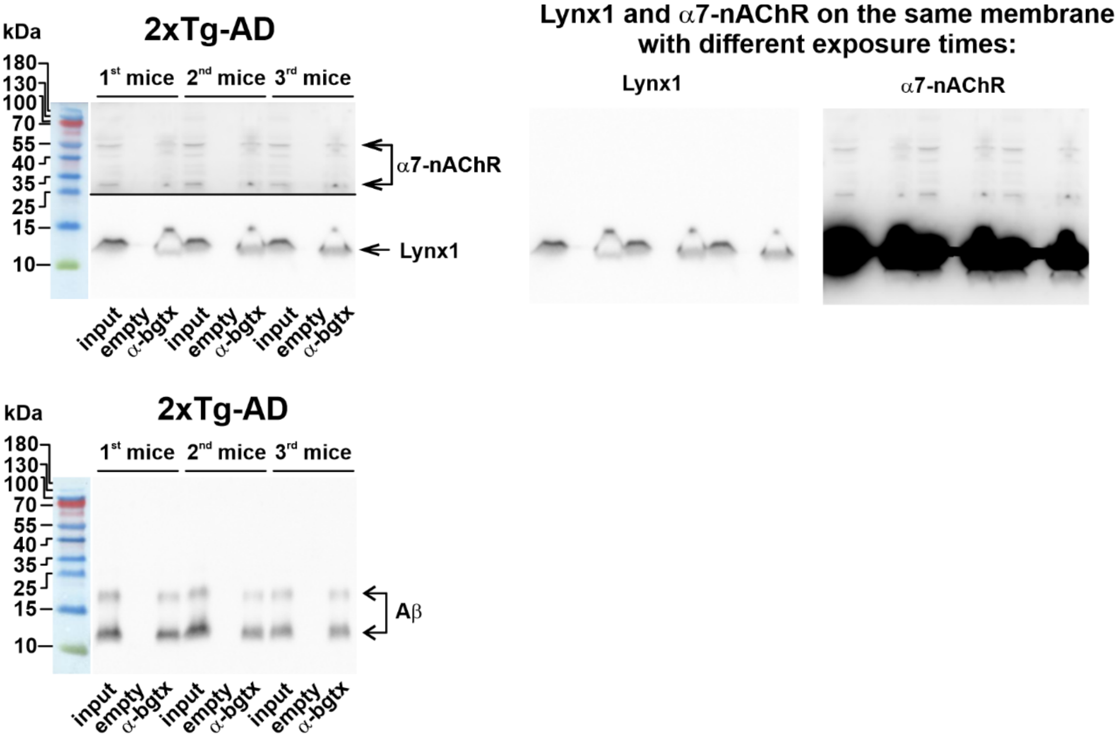

Figure S4. The whole Western-blotting membranes showing the α7-nAChR partners in the cerebellum of 2xTg-AD mice by affinity extraction using NHS-Sepharose resin coupled with α-Bgtx (n = 3). The empty resin blocked by 500 mM ethanolamine +5% skim milk was used as a negative control.

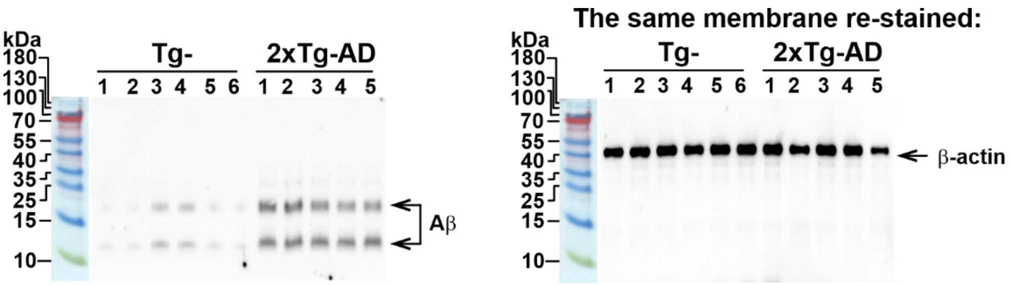

Figure S5. The whole Western blotting membranes showing the expression of Aβ1-42 in the cerebellum of Tg^- and 2xTg-AD mice.

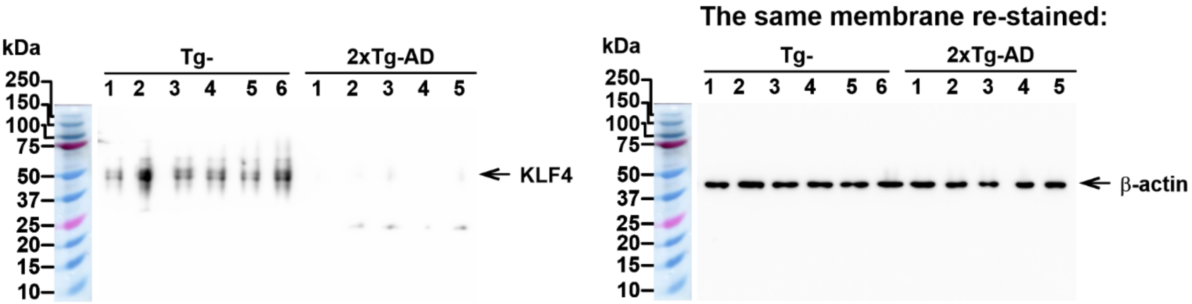

Figure S6. The whole Western blotting membranes showing the expression of KLF4 in the cerebellum of Tg^- and 2xTg-AD mice.

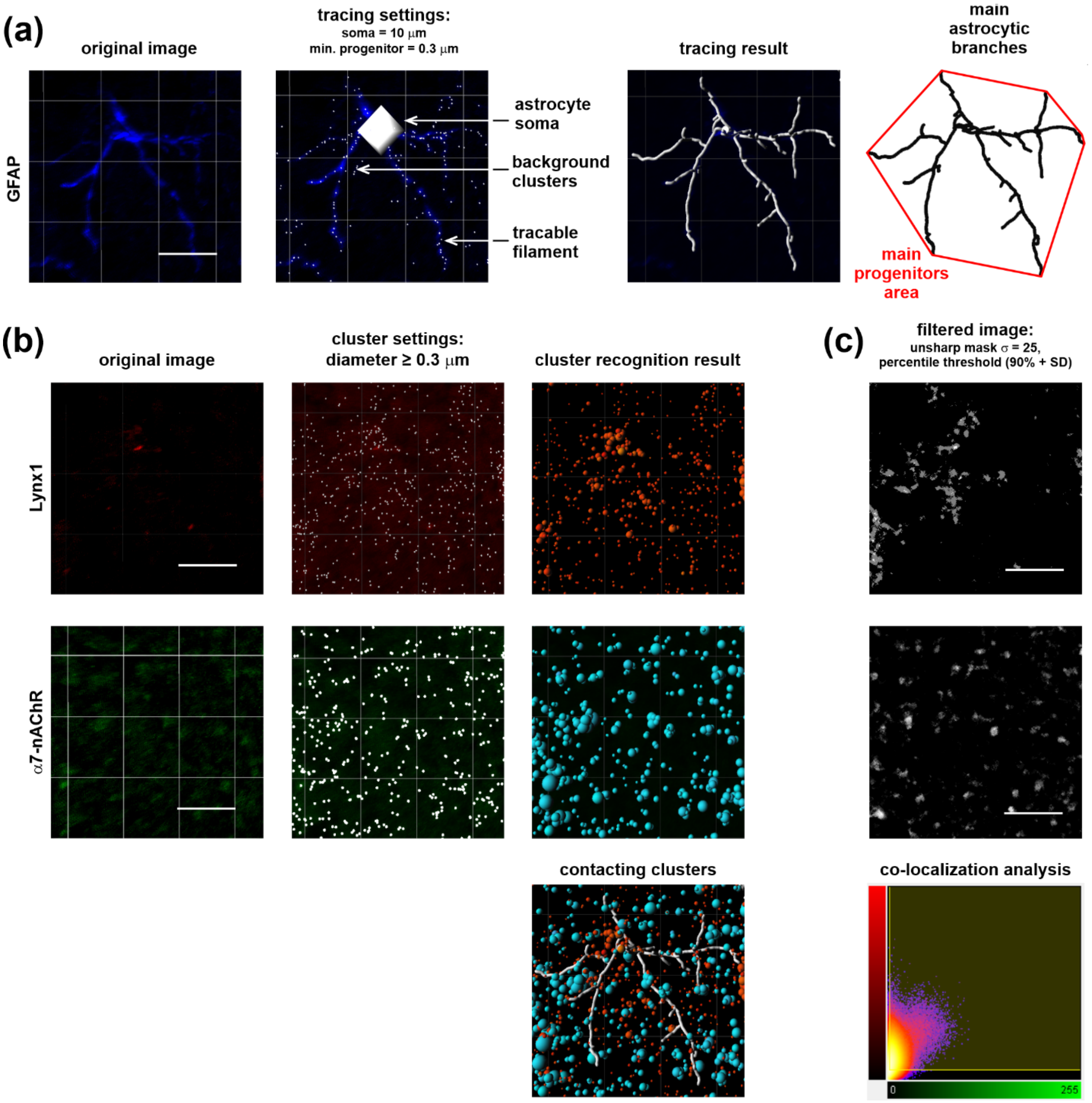

Figure S7. The workflow for image analysis: (a) the astrocyte reconstruction scheme; (b) the analysis of α7-nAChR and Lynx1 clusters in 3D images; (c) assessment of α7-nAChR and Lynx1 clusters co-localization using the Pearson's regression, scale = 10 μm.

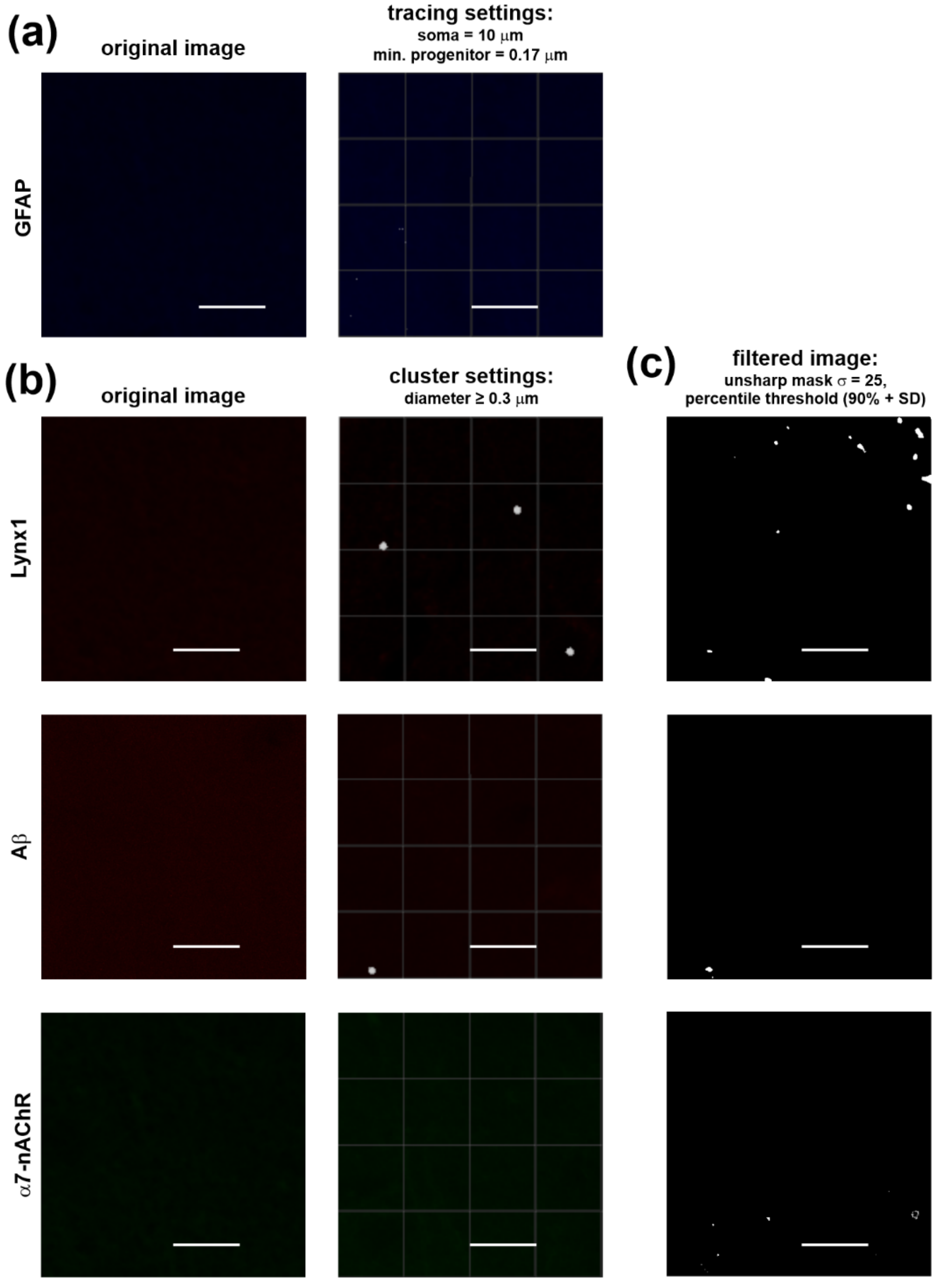

Figure S8. The cerebellum slices stained only by secondary antibodies: (a) astrocytic reconstruction; (b) analysis of Lynx1, Aβ, and α7-nAChR clusters in 3D image; (c) clusters after image filtering, scale = 10 μm.
